# Assessing the health of the gut microbial organ: why and how?

**DOI:** 10.1172/JCI184313

**Published:** 2025-06-02

**Authors:** Orlando DeLeon, Eugene B. Chang

**Affiliations:** Department of Medicine, Section of Gastroenterology, Hepatology, & Nutrition, University of Chicago, Chicago, Illinois, USA.

## A failure to translate findings

The gut microbiome is the complex collection of microorganisms residing within our intestinal tracts, including native bacteria, fungi, and viruses (1). Research over the past several decades has increased the scientific, medical, and general public’s appreciation of the gut microbiome’s importance in human health to a degree equivalent to other essential organs of the body (2). The topics in the series of review articles associated with this Perspective similarly reflect the extensive impact of the microbiome in the gut and beyond, ranging from the role of diet in gut microbiome composition; the perinatal microbiome’s impact on neonates and long-term outcomes in offspring; the microbiome in diseases of the skin, airways, and gastrointestinal tract; in cancer; and in vaginal health.

It’s clear that we cannot live healthy lives without an equally healthy gut microbiome (3, 4). Toward this end, a wide array of microbiome-based and -directed products are marketed as nutraceuticals that claim to have health and medicinal benefits, but few, if any, actually have supporting scientific or clinical evidence for efficacy or federal certification or approval (5, 6). Still, a plethora of experimental data unambiguously demonstrates the importance of the gut microbiome in human health and its massive potential as a therapeutic target. This lack of progress toward new FDA-approved microbiome-based drugs has been a great challenge and burden to the field, stymieing enthusiasm and confidence in the nascent field of microbiome medicine. We as stakeholders in the field have fallen short in our responsibilities as biomedical researchers with translating our research into therapies that can directly improve human health. Therefore, we must ask ourselves as clinicians, doctors, and scientists, despite decades of extensive research investigations that have resulted in a deeper understanding of the physiological importance of the gut microbiome, why has the translation of this knowledge to clinical practice lagged so far behind? What have been the challenges and bottlenecks in moving the field of microbiome medicine forward?

## The gut microbial organ lacks health metrics

The gut microbiome is a bona fide vital organ of the human body, providing essential functions that are required for developmental processes, protection against pathogens, immunity, metabolism, and other functions critical to maintaining host health (7). Unlike other vital organ systems of the body, the gut microbiome is acquired, initially, from vertical transfer of maternal gut microbes and then from other individuals later in life (8). We now recognize that each region of the bowel is a unique ecosystem that has its own assembly rules and conditional requirements whereby specific gut microbiota that are fit can stably engraft and provide functions necessary and essential for its host (9, 10). This is not a random process and is likely the result of eons of cospeciation or coevolution between host and microbe to achieve states of phylosymbiosis, i.e., the phenomenon where the relationships of microbial communities within a host mirror the evolutionary (phylogenetic) relationships of the host species (11, 12). Not surprisingly, nonhuman-derived gut microbiota, e.g., many probiotics derived from environmental, dairy, and bovine sources, are not fit or capable of engraftment in humans, accounting for the lack of their efficacy (13).

Like other organ systems, dysfunction or injury to the gut microbiome (a condition called “dysbiosis”) can cause or contribute to many pathologies and diseases, e.g., immune disorders like inflammatory bowel diseases, cancer, aging, neurogenerative disorders (dementia, movement disorders), metabolic diseases that include diabetes, obesity, metabolic syndrome, and metabolic dysfunction-associated fatty liver disease (14). In these cases, restoration of a healthy gut microbiome (a condition called “eubiosis”) can, in theory, prevent, lower the risk of, or lead to improvement and cure of these disorders (15). While this gives promise to the future of precision microbiome medicine, the field has not substantially advanced and continues to be mired in empiricism. This is because, unlike other vital organ systems of the body for which informative diagnostic and management tools are part of daily medical practices, there are currently no reliable tests that allow us to assess whether the gut microbiome is healthy or unhealthy. Development of a tool to assess the health state of the microbiome would be a game changer for diagnosis; clinical management of diseases; informing therapeutic timing and responses; assessing efficacy of microbiome-based interventions (MBIs), including fecal microbiota transplant (FMT) efficacy; evaluating effectiveness of diet; deterrence of multidrug resistant (MDR) pathogens; and stratifying disease for individual, precision medicine.

## Current approaches to define gut microbiome health

**Figure 1:**
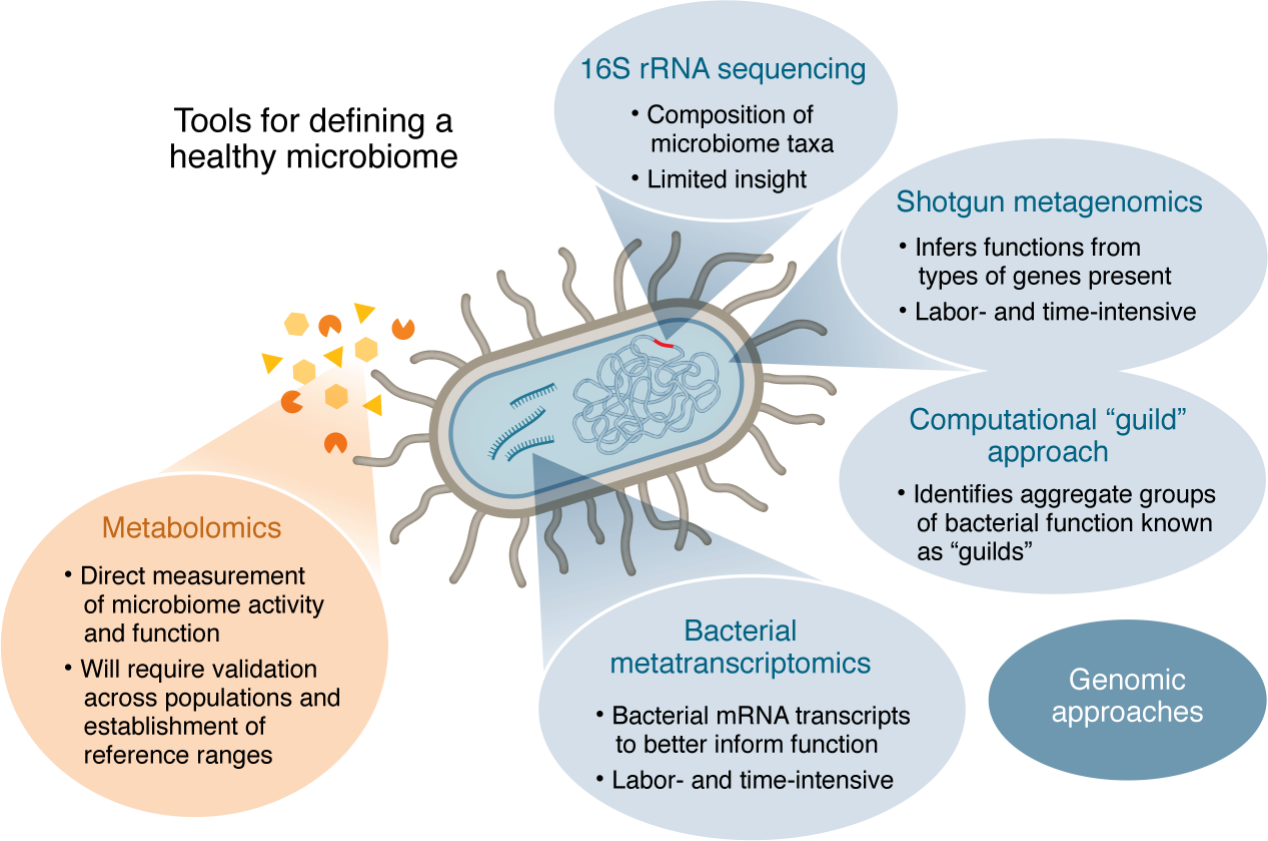
Tools for defining a healthy microbiome.

Current approaches used to define gut microbiome health have been largely based on genomic assessments of the microbiome (Figure 1) (16). The most common of these are 16S rRNA sequencing and shotgun metagenomic sequencing. Attempts to use these technologies to define health have been limited in their success largely due to the heterogeneity of microbiota across individuals and populations (17, 18). 16S rRNA sequencing, which sequences portions of the bacterial ribosomal gene, only provides information on microbiome composition (bacterial taxa and the proportions of each). Shotgun metagenomics, which sequences the entirety of the DNA extracted from microbiome samples (e.g., stool), can be used to infer function by measuring the types of genes. For example, studies quantifying the number and proportions of carbohydrate-degrading enzymes (CAZymes) have reported their correlation with increased nutrient extraction and digestion, which can impact metabolism (19). Population studies have shown that functional potential of the gut microbiome is relatively homogenous between individuals and populations, suggesting that the specific human bacterial strain is less important than the functions it provides and that many types of microbes across all individuals can perform a common set of functions (18). Bacterial transcriptomics (metatranscriptomics that is based on sequencing of microbiota mRNA transcripts) is a step closer to approximating the functional output of the microbiome, but suffers from similar deficiencies as shotgun sequencing in time and labor, annotation, and is quite technically difficult to perform due to the labile nature of bacterial RNA (20). Moreover, several nuances of these approaches limit their usefulness for clinical application, including the laborious nature for preparing and sequencing samples, the cost and expertise needed for data analysis without any guarantee that the inferences made from the genes translate to actual production or real function, and the limited annotation of these genes, as fewer than 50% of genes in a typical metagenome can be identified (21). Additionally, host DNA contamination in regions of low bacterial load, such as the small intestine, can complicate interpretations. Still, there have been some promising advances made through bioinformatic analysis of metagenomic sequences to identify functional “guilds,” groups of bacteria with a common aggregate function, that can gauge the health of the gut microbiota (22). This approach has both rationale and promise, but is still largely a research tool.

## Developing clinically useful tools to quantify microbiome health

The health state of the gut microbial organ is best measured through direct and functional approaches, rather than the taxonomical-based approaches that are widely used by much of the field. We opine that metabolomics-based tools and approaches, as opposed to genomics-based methods, best fit these criteria and can be readily adapted for clinical application. Rather than inferring function from taxonomy or gene sequences, the small molecules produced or modified by the microbiome represent end products of bacterial metabolism and thus are closest to fully encompassing the total functional output of the microbiome (23). Many of these small molecules are biologically active or used by our bodies directly, directly representing microbiome function (e.g., production of short chain fatty acids or vitamins) (24). Metabolites used in a functional test to assess the health of the microbial organ must be representative of key functional subsystems of a healthy gut microbiome. While ongoing research is rapidly uncovering the roles of microbiome metabolites, a large number of metabolites have already been well studied (25, 26). For example, sustained levels of butyrate are considered important products of proper microbial fermentation and provide many important functions than include providing energy, immune regulation, maintenance of gut barrier maintenance, and mucosal healing (27, 28). Any functional test should address many, if not all, of these microbiome functions.

In our view, a clinically useful tool for measuring the function and health state of the gut microbiome would be developed according to the guidelines outlined here. First, biologically relevant metabolites discovered by untargeted methods should be identified and validated with absolute quantification using known standards to insure rigor and reproducibility. Second, we must develop normal reference ranges for these metabolites in healthy human populations much like we have for blood chemistries. We must understand the optimal, healthy population distribution of metabolites representative of these specific microbiome functions, and at which point increasing or decreasing levels (excessive function or lowered function) can negatively impact health. Third, these reference ranges must be studied across multiple human populations to determine whether a singular group of metabolites are present universally in diverse healthy individuals or whether population-specific metabolite ranges are required. Fourth, while humans have relatively stable microbiomes during adulthood, they vary rapidly during early childhood development and during old age, which may require different metrics and a different panel and ranges of metabolites. Fifth, these tools need to be clinically validated for rigor and reproducibility. Efforts to collect these data can be greatly enhanced by more convenient methods of sample collection that do not rely on in-person clinical visits or require the direct handling of stool or refrigeration. Feasible and clinical utility for approaches developed in alignment with these guidelines were shown with the development of targeted Mass Spectrometry–based quantitative prototype metabolite panels, where normal reference ranges for each panel marker have been developed from discovery and validation cohorts (29). Future prototypes can then be optimized to be demographic- and population-specific, and advanced artificial intelligence (AI) could be incorporated to enhance informational value, affordability, turnaround time, and personalized recommendations.

The technologies currently exist to develop future diagnostic, predictive, and management tools for assessing the health of the gut microbial organ. What is left to address is the dedicated time, international cooperation, and investment towards this singular goal. However, doing so will represent the biggest advance in microbiome medicine since the development of high-throughput sequencing. We will finally be able to answer the question “what is a healthy microbiome?”, quantify that health, and drive the rapid development of precision therapeutics.

## Version 1 06/02/2025

Electronic publication
